# Investigation of the Efficiency of Shielding Gamma and Electron Radiation Using Glasses Based on TeO_2_-WO_3_-Bi_2_O_3_-MoO_3_-SiO to Protect Electronic Circuits from the Negative Effects of Ionizing Radiation

**DOI:** 10.3390/ma15176071

**Published:** 2022-09-01

**Authors:** Artem Kozlovskiy, Dmitriy I. Shlimas, Maxim V. Zdorovets, Elena Popova, Edgars Elsts, Anatoli I. Popov

**Affiliations:** 1 Engineering Profile Laboratory, L. N. Gumilyov Eurasian National University, Satpaev Str. 5, Nur-Sultan 010008, Kazakhstan; 2 Laboratory of Solid State Physics, The Institute of Nuclear Physics, Ibrag and ov Str. 1, Almaty 050032, Kazakhstan; 3 Centro de Investigación en Astronomía, Universidad Bernardo O’Higgins, Santiago 8370854, Chile; 4 Institute of Solid State Physics, University of Latvia, LV-1063 Riga, Latvia

**Keywords:** protective materials, telluride glasses, shielding, microelectronics, gamma radiation

## Abstract

This article considers the effect of MoO_3_ and SiO additives in telluride glasses on the shielding characteristics and protection of electronic microcircuits operating under conditions of increased radiation background or cosmic radiation. MoO_3_ and SiO dopants were chosen because their properties, including their insulating characteristics, make it possible to avoid breakdown processes caused by radiation damage. The relevance of the study consists in the proposed method of using protective glasses to protect the most important components of electronic circuits from the negative effects of ionizing radiation, which can cause failures or lead to destabilization of the electronics. Evaluation of the shielding efficiency of gamma and electron radiation was carried out using a standard method for determining the change in the threshold voltage (∆U) value of microcircuits placed behind the shield and subjected to irradiation with various doses. It was established that an increase in the content of MoO_3_ and SiO in the glass structure led to an increase of up to 90% in the gamma radiation shielding efficiency, while maintaining the stability of microcircuit performance under prolonged exposure to ionizing radiation. The results obtained allow us to conclude that the use of protective glasses based on TeO_2_–WO_3_–Bi_2_O_3_–MoO_3_–SiO is highly promising for creating local protection for the main components of microcircuits and semiconductor devices operating under conditions of increased background radiation or cosmic radiation.

## 1. Introduction

One of the leading and most important areas of research in the field of microelectronics is the study of the resistance of microelectronic devices and semiconductor devices to radiation, as well as the assessment of their degradation under operating conditions when exposed to ionizing radiation and increased background radiation [1,2]. This area of research has been of high relevance in recent years due to the increase in the number of manufactured devices and their areas of application, with the transition of most industries to robotization, as well as the complications and reduction in the geometric dimensions of microcircuits and semiconductor devices, which requires the abandonment of most traditional methods of protection against the negative effects of ionizing radiation [3,4,5]. This problem is especially acute in space technology and nuclear power engineering and when operating instruments under conditions of exposure to radiation, particularly with high-energy electrons, gamma radiation, protons or heavy ions [6]. At the same time, due to its nature, ionizing radiation can lead to destabilization of the onboard equipment of spacecraft and satellites, both as a result of the cumulative effect of the absorbed dose during prolonged exposure to ionizing radiation, and in the case of single effects that are of a probabilistic nature [7]. Exposure to ionizing radiation can lead to parametric failure, and to the occurrence of single failures and breakdowns in dielectric layers that occur during the initialization of the processes of radiation-stimulated chemical reactions. Side effects caused by electromagnetic or thermal effects may also occur, resulting in malfunctions or thermomechanical stresses and distortions. At the same time, understanding the processes of interaction of ionizing radiation on semiconductor devices, as well as microelectronic devices, is very important for the design and development of electronic devices, and therefore, much attention has been paid to this area in recent years [8,9,10]. Note that while the processes of radiation damage are fairly well understood in binary oxides and halides [11,12,13,14,15,16,17], the situation in multicomponent glasses is still far from detailed [18,19,20].

One of the solutions to the problem of radiation damage is the use of local protection against ionizing radiation of the most vulnerable components of microcircuits or semiconductor devices, using protective coatings or glasses with a high absorbing ability, which makes it possible to either reduce the intensity of ionizing radiation or completely absorb it [21,22,23,24,25]. The choice of telluride glasses doped with various oxide components as protective materials makes it possible to obtain high-strength, optically transparent materials with a high absorption capacity, which enables the reduction of the intensity of ionizing radiation by several orders of magnitude, thereby significantly increasing the level of protection against the negative effects of radiation [26,27]. The potential of telluride glasses for shielding gamma and neutron radiation in a wide energy range has been reported in a number of works, and in most of them the concept of creating protective shielding materials is based on an increase in the *Z_eff_* value, a characteristic that is responsible for the absorbing ability of the material, due to the fact that the absorption of gamma radiation is directly dependent on the given value of the material in which braking occurs [28,29]. However, for microelectronics, especially those used in spacecraft, the use of classical lead-based shielding materials is not advisable due to their large mass, which leads to an increase in the overall dimensions and weight of microelectronic devices [30,31,32,33,34,35]. In view of this, the main aim of this work is to assess the prospects of using telluride glasses doped with MoO_3_ and SiO to create local protection against the ionizing radiation of microelectronic devices by depositing thin glasses with high absorption capacity and screening characteristics on the most important nodes. The novelty of this study lies in assessing the possibility of using telluride glasses as protective materials for microelectronic devices, as well as assessing the possibility of creating local protection against the negative effects of ionizing radiation.

## 2. Experimental Methods

Synthesis of TeO_2_–WO_3_–Bi_2_O_3_–MoO_3_–SiO glasses was carried out using the standard technology for obtaining amorphous glasses based on tellurium and its compounds [33,34,35]. The process of obtaining glasses consisted in weighing the initial powders in a given stoichiometric ratio, after which the resulting mixtures were subjected to mechanochemical mixing until a homogeneous mixture was obtained. After stirring, the resulting mixtures were placed in heat-resistant crucibles for subsequent melting in a muffle furnace at a temperature of 1000 °C for 1 h. During the melting process, the mixture was subjected to stirring in order to eliminate the bubbles that occur during melting. After melting, the resulting mixture was annealed at a temperature of 500 °C in order to subsequently pour the samples into molds.

The following oxides were used for synthesis: tellurium dioxide (TeO_2_), tungsten trioxide (WO_3_), bismuth(III) oxide (Bi_2_O_3_) molybdenum(VI) oxide (MoO_3_), and silicon monoxide (SiO) in given stoichiometric ratios. All reagents used were purchased from Sigma Aldrich (St. Louis, MO, USA); chemical purity was 99.95%. The choice of components for synthesis was based on a priori data on the properties of the selected oxides. The main components of the glasses were TeO_2_ and WO_3_, whose density and absorbent characteristics form the basis of protective glasses and ceramics used for shielding ionizing radiation. Thus, the addition of Bi_2_O_3_ to the composition of the glasses was due to its protective properties, which make it possible to reduce the sintering temperature and increase the melting rate of the components. The choice of SiO was due to its dielectric properties, which make it possible to increase the resistance of glasses to electrical breakdowns [36,37]. Silicon monoxide was also chosen for its chemical properties (resistance to oxidation processes when heated) and melting point. The choice of MoO_3_ as a dopant was due to its anti-corrosion properties, which make it possible to increase the resistance of materials to corrosion and degradation. MoO_3_ is also used in the production of photochromic mirrors or light-redistributing filters [38,39,40].

Table 1 presents the data on the content of the components used for the synthesis of the glasses, as well as their designations. These data are used further in describing the observed effects and the results of experimental studies. The density of the studied glasses was determined by the Archimedean method, in view of the amorphous nature of the glasses, which did not allow estimation of the density by X-ray methods.

**Figure 1:**
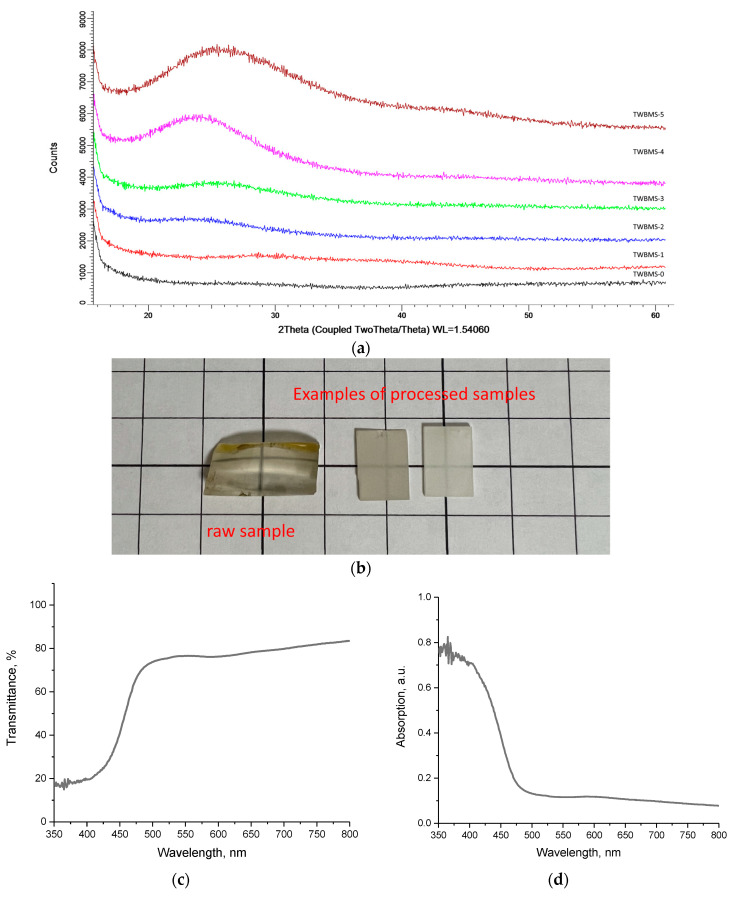
(**a**) Results of X-ray phase analysis; (**b**) examples of images of obtained glasses before and after processing; (**c**) results of optical measurements of transmission spectra; (**d**) results of optical measurements of absorption spectra.

The glasses were obtained in two stages. The first stage consisted in weighing the initial components with a given content and subsequent mechanochemical grinding in a PULVERISETTE 6 planetary mill (Fritsch international, Idar-Oberstein, Germany) for 1 h at a grinding speed of 400 rpm. For grinding, a glass and grinding balls with a diameter of 10 mm made of tungsten carbide were used; these did not introduce impurities into the ground mixture during the grinding process, as confirmed using the energy-dispersive analysis method. The second stage of glass fabrication consisted in thermal sintering and subsequent quenching of ground mixtures in a SNOL muffle furnace at a temperature of 1000 °C for 1 h. The samples obtained were amorphous glasses with sufficient transparency and resistance to mechanical shocks and damage. According to the data of the X-ray phase analysis, it was found that the synthesized glasses had an amorphous nature (see data in Figure 1).

The images in Figure 1c,d show the results of measuring the optical properties of the synthesized transmission and absorption glasses (see data in Figure 1c,d).

Testing of the shielding characteristics was carried out in several stages, consisting in a series of experiments aimed at determining the effectiveness of reducing the intensity of gamma radiation in the energy range of gamma rays from 130 keV to 1270 keV. This range was obtained using three sources of gamma radiation” Co^57^, Cs^137^ and Na^22^. All five experiments to determine the shielding efficiency were performed in parallel in order to determine the measurement error and the stability of the preservation of indicators for different series. The choice of this energy range of gamma radiation was due to its ability to simulate the processes of interaction between radiation and matter, taking into account the photoelectric effect, the Compton effect and the formation of electron–positron pairs. All three types of interaction between gamma radiation and matter have pronounced energy dependences, which allow coverage of the energy range used. The amount of shielding or reduction in the intensity of gamma radiation was estimated using Formula (1):

(1) $$RFE=\left(1-\frac{I}{I_{0}}\right)\times 100\%$$

where *I* and *I*_0_ are the intensities of gamma radiation before and after shielding that passed through the protective shield and were recorded by the NaI detector.

Linear and mass coefficients were estimated using Formulas (2) and (3).

(2) μ=lnI0Id

(3) $$\mu_{m}=\frac{\mu }{\rho }$$

where *I*_0_ is the value of the initial intensity, *I* is the value of the intensity after shielding; *d* is thickness, and *ρ* is the glass density.

The value of the effective atomic number (*Z_eff_*) was calculated using Formula (4):

(4) $$Z_{eff}=\frac{\sum_{i}f_{i}A_{i}{\left(\mu_{m}\right)}_{i}}{\sum_{i}f_{i}\frac{A_{i}}{Z_{i}}{\left(\mu_{m}\right)}_{i}}$$

where *f_i_* is the ratio of number of atoms of the element *i* to the total number of atoms, *A_i_* is the molar mass, and *Z_i_* is the atomic number of the element *i.*

The shielding scheme looked as follows: a NaI detector was placed at a distance of 10 cm from the gamma ray source, which recorded the radiation intensity. A sample of a protective shield made of glass with a thickness of *d* = 1, 1.5, and 2 mm was placed in front of the detector. To collect statistics, the duration of the shielding experiments was 2 h.

Testing of the shielding characteristics of the synthesized glasses to protect electronic microcircuits from the negative effects of ionizing radiation was carried out by determining the magnitude of the change in the threshold voltage (∆*U*) when measuring the current–voltage characteristics of microcircuits placed behind the shielding glass. Testing was carried out for two types of ionizing radiation: gamma rays with an energy of 1.3 MeV and electrons with an energy of 1.0 MeV in the dose range of 50–500 kGy. The accumulated dose was determined using standard ionizing radiation detectors placed next to the microchips to control the dose load. Glasses with a thickness of 1 mm were used as protective materials.

## 3. Results and Discussion

**Figure 2:**
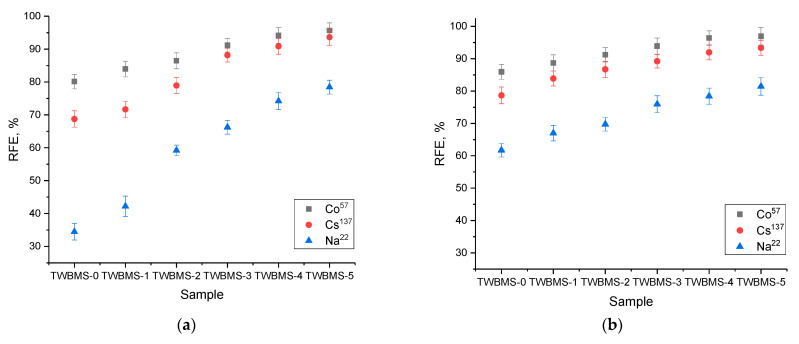

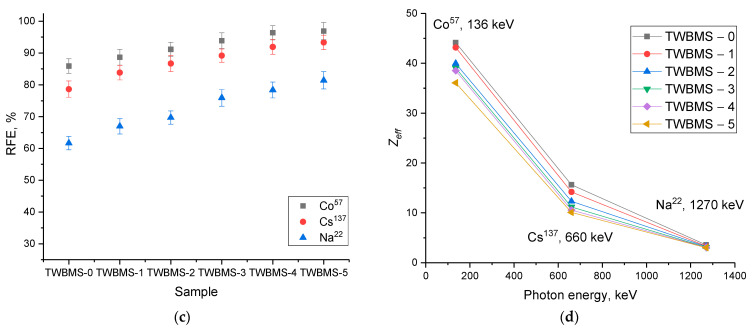
Results of RFE calculations depending on glass thickness: (**a**) 1.0 mm; (**b**) 1.5 mm; (**c**) 2.0 mm; (**d**) the results of calculating the value of *Z_eff._*

Figure 2 shows the evaluation results of the efficiency of reducing the intensity of gamma radiation using synthesized glasses, depending on the composition of the glasses as well as their thickness. The calculation was carried out using Formula (1). As can be seen from the data presented, in shielding against gamma radiation from the Co^57^ source (130 keV), the change in the glass composition made a greater contribution than the increase in glass thickness, and in the cases of the TWBMS–4 and TWBMS–5 glasses, an increase in thickness from 1 mm to 2 mm did not lead to a significant increase in absorption efficiency (less than 1%). At the same time, changing the composition of glasses by adding MoO_3_ and SiO to them led to an increase in shielding efficiency from 80–85% to 93–95%, depending on the thickness. This effect was due to the absorption capabilities of MoO_3_ and SiO, as well as a change in the *Z_eff_* value, which had a direct effect on the shielding efficiency. 

In the case of gamma radiation from a Cs^137^ source (660 keV), the influence of glass composition was more pronounced for the thinnest sample (1 mm) and less pronounced for thicknesses of 1.5 mm and 2 mm. For a given energy of gamma rays, mechanisms of interaction with matter by Compton scattering are most likely, and are also significantly dependence on the *Z_eff_* value. In this case, an increase in the *Z_eff_* value due to a change in the content of glass components led to an increase in the absorption and attenuation of gamma radiation due to a change in the electron density and an increase in the effective cross section. At the same time, for thin glasses, the difference between the shielding value for glasses that did not contain MoO_3_ and SiO and that for glasses with a concentration of these components of 7.5–10% was more than 15%, while an increase in thickness led to a decrease in this difference to 7–10%. This difference was due to an increase in the absorption capacity of glasses in the case of the dominance of Compton scattering processes.

In the case of gamma radiation shielding from a source of Na^22^ (1270 keV) for TWBMS–0 and TWBMS–1 glasses with a thickness of 1 mm, the shielding efficiency was less than 50%, which indicates that most of the gamma radiation passed unhindered through the glasses. At the same time, for glasses containing MoO_3_ and SiO, the shielding efficiency increased, which was due to the fact that in the case of high-energy gamma rays, the main interaction process was the formation of electron–positron pairs, the probability of which was proportional to Z^2^. In the case of an increase in glass thickness from 1.0 mm to 1.5 mm, the efficiency for samples TWBMS–0 and TWBMS–1 more than doubled, but a further increase in thickness did not lead to a similar effect.

For a glass thickness of 2.0 mm, a change in the glass composition due to an increase in the concentration of MoO_3_ and SiO for screening gamma rays with energies of 130 and 660 keV, the shielding efficiency was practically unchanged and amounted to more than 90%. In the shielding of gamma rays with an energy of 1270 keV, an increase in the concentration of MoO_3_ and SiO led to an increase in shielding efficiency from 70% to 83–85%. Such a difference in shielding is due to the processes of interaction between gamma quanta and matter, depending on the initial energy. At low energies of gamma radiation, the main interaction processes are the photoelectric effect and Compton scattering, for which the formation of secondary radiation is unlikely, and the cross section of the interaction reaction has a pronounced dependence on *Z_eff_*. In the case of gamma rays with energies above 1 MeV, the main processes of interaction are the formation of electron–positron pairs, which are accompanied by a high probability of the formation of secondary radiation. In this case, the presence of various elements in the structures of the shielding materials leads to an increase in absorption, which reduces the intensity of radiation and also partially extinguishes it. However, this effect is not linear with increases in the concentration of components in glasses, and has a complex dependence on the types of glass components used and their percentages.

Figure 2d shows the results of changing the value of *Z_eff_* depending on the energy of shielded gamma rays, calculated according to the methodology proposed in [27,33,40,41,42,43,44,45,46,47].

In the course of the study, it was found that with the addition of the MoO_3_ and SiO dopant, a decrease in density was observed, while the evaluation of the Zeff value showed that this value did not change significantly, in view of the fact that with the addition of MoO_3_ and SiO, an increase in the linear and mass absorption coefficients was observed, a change that indicated an increase in absorption efficiency. In view of this, the absorption efficiency depended not only on the *Z_eff_* value, but as the results of a number of experimental studies show, an increase in absorption efficiency was due to the synergistic effect of absorption due to a large number of different ions in the glass structure and the presence of a large number of absorbing centers capable of reducing the intensity of passing gamma rays as well as that of the resulting secondary radiation. Similar effects were observed in a number of works [25,26,27,28,29,30,31,32,33,34,35,36,37,38,39,40] focusing on the effects associated with changes in the elemental composition of glasses due to the variation of the components, which, in turn, affects the absorption capacity. These effects are associated with a change in the electron density distribution, as well as differences in the atomic radii and their masses of the elements of the glass components, which, when interacting with gamma radiation, play a very important role in absorption. An analysis of the optical absorption and transmission spectra of the glasses showed that the addition of MoO_3_ and SiO led to an increase in absorptivity, as well as a shift in the fundamental absorption edge, which indicated a change in the electron density of the glasses with an increase in the concentration of dopants.

Furthermore, the addition of MoO_3_ and SiO components led to a 30–40% increase in the mechanical strength of the glass samples compared to glasses that did not contain these dopants. Such a change is associated with the effect of strengthening materials when silicon oxide is added to them, which in turn leads to an increase in the resistance of glasses to external mechanical influences, and also expands the possibilities of their practical application under conditions with possible mechanical pressures or shocks.

**Figure 3:**
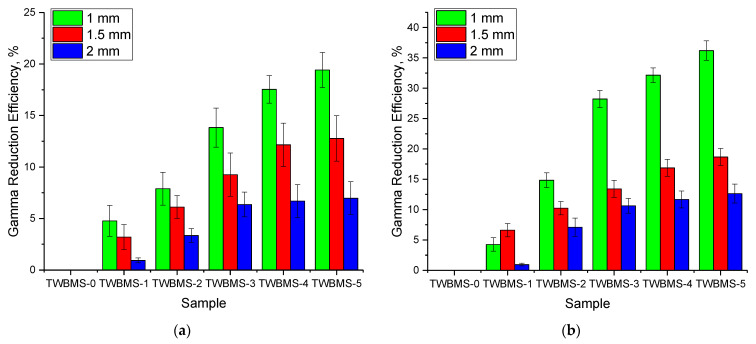

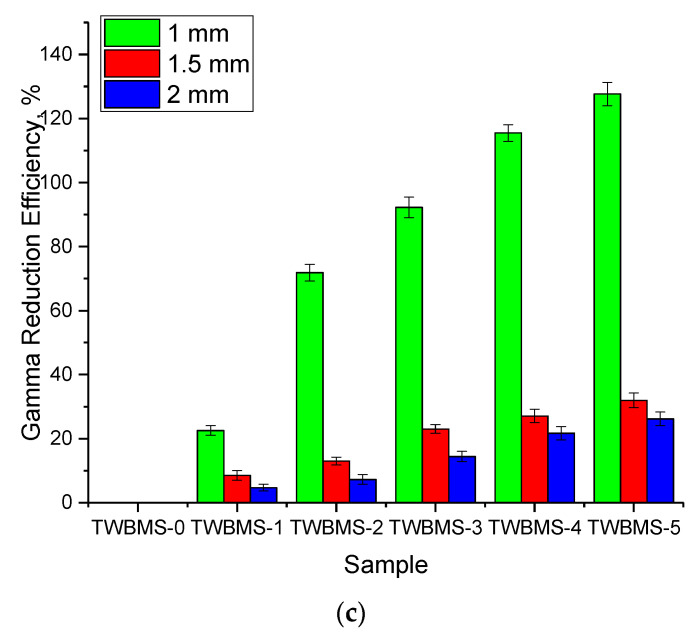
Diagrams of the change in the absorption efficiency of the gamma radiation intensity in comparison with a sample not containing MoO_3_ and SiO: (**a**) when shielding gamma rays with an energy of 130 keV; (**b**) when shielding gamma rays with an energy of 660 keV; (**c**) when shielding gamma rays with an energy of 1270 keV.

The same situation was observed with an increase in the thickness of shielding glasses. As can be seen from the data presented in Figure 3 of the dependences of the change in the gamma radiation absorption efficiency in comparison with the sample not containing MoO_3_ and SiO, it was found that the greatest increase in shielding efficiency was observed for 2.0 mm thick samples in shielding gamma rays with an energy of 130 keV. In shielding of gamma rays with energies of 660 and 1270 keV, an increase in the concentrations of MoO_3_ and SiO from 7.5% to 10% did not lead to a sharp increase in efficiency relative to each other, while in relation to the original sample, the increase in efficiency was more than 10–20%.

The efficiency of gamma radiation absorption was estimated using formula (4):

(5) $$GRE=\left(\frac{RFE_{i}-RFE_{0}}{RFE_{0}}\right)\times 100\%$$

where *RFE_i_* and *RFE*_0_ are intensity reduction efficiencies for samples with and without dopants.

When shielding gamma rays with an energy of 130 keV, the increase in efficiency, depending on the concentration of MoO_3_ and SiO, was no more than 2–7% with increasing glass thickness, which was due to the fact that in shielding of low-energy gamma rays, the efficiency value was more than 80% for all thicknesses. Thus, it was established that glasses with a thickness of 1.0 mm were most effective in shielding of low-energy gamma rays, as were multiphase glasses in shielding of high-energy gamma rays.

**Figure 4:**
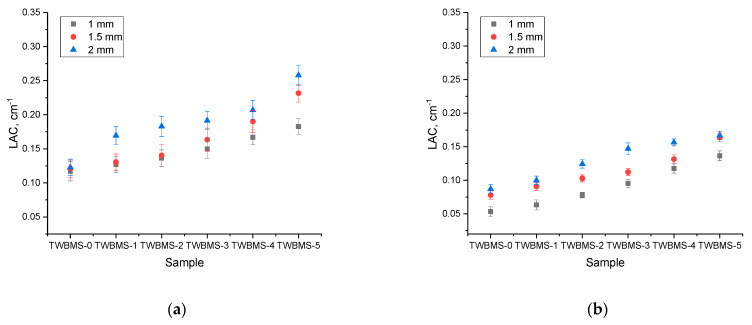

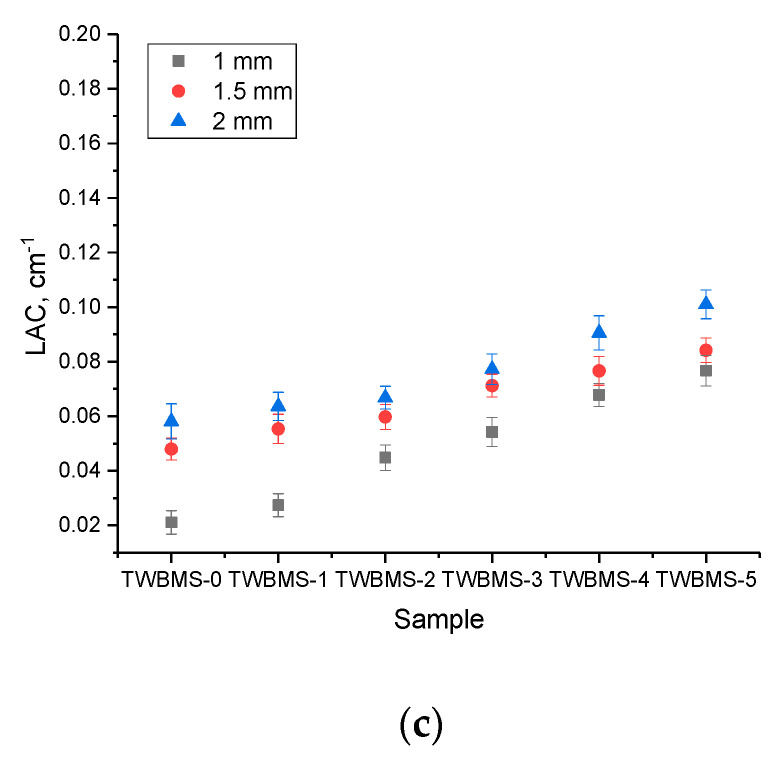
Results of evaluation of the change in the linear attenuation coefficient of gamma radiation depending on the type of gamma-quantum source: (**a**) Co^57^, 130 keV; (**b**) Cs^137^, 660 keV; (**c**) Na^22^, 1270 keV.

**Figure 5:**
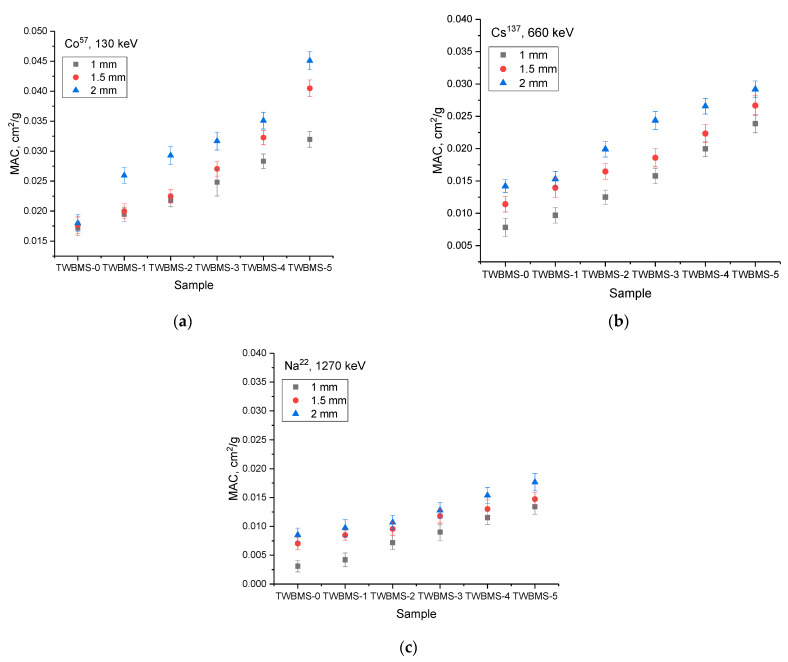
Results of evaluation of the change in the mass attenuation coefficient of gamma radiation depending on the type of gamma-quantum source: (**a**) Co^57^, 130 keV; (**b**) Cs^137^, 660 keV; (**c**) Na^22^, 1270 keV.

Figure 4 and Figure 5 show the dependences of the change in the coefficient of linear and mass attenuation of gamma radiation with different energies for the different thicknesses of the protective glasses under study.

The general view of the presented trends in the change in the linear and mass attenuation coefficients indicate that a change in the composition of the glasses led to an increase in the gamma radiation absorption efficiency, as well as a decrease in its efficiency by more than 2–2.5 times compared to glasses that did not contain MoO_3_ and SiO in the case of low-energy gamma quanta (Co^57^, 130 keV). Moreover, in the TWBMS–4 and TWBMS–5 samples, the absorption reduction efficiency increased more significantly. 

**Figure 6:**
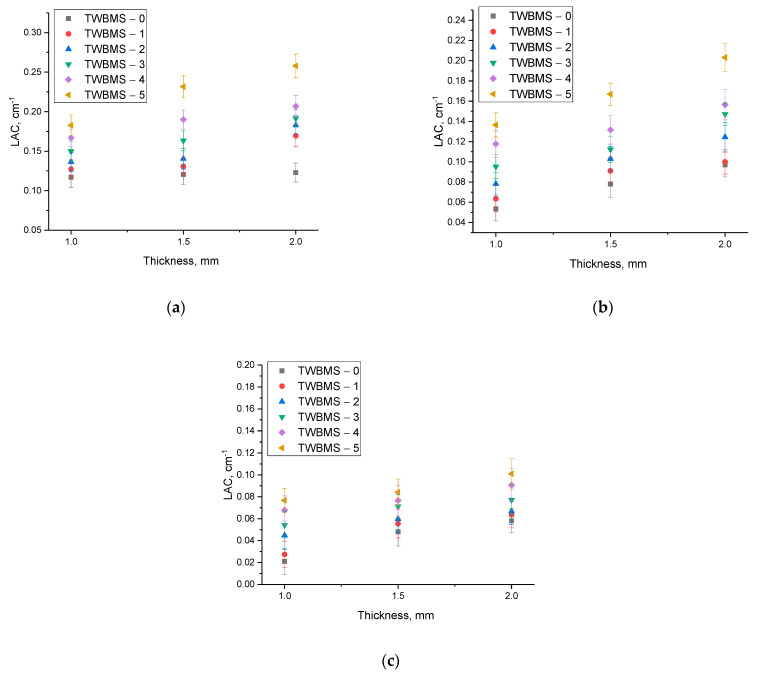
Changes in the LAC value depending on the thickness of the glasses:(**a**) Co^57^, 130 keV; (**b**) Cs^137^, 660 keV; (**c**) Na^22^, 1270 keV.

Figure 6 shows the dependences of the shielding efficiency estimated using the linear attenuation coefficient, depending on the thickness of the protective glasses.

As can be seen from the presented data, an increase in the glass thickness led to an increase in the shielding efficiency, which can be explained quite simply: the thicker the protective coating, the more efficiently the ionizing radiation was absorbed into it. At the same time, as can be seen from the data in Figure 6, this effect was most pronounced for gamma rays with an energy of 1270 keV with glasses that contained dopants. Such a change in the linear attenuation coefficient was due to the fact that at high energy levels of gamma radiation, the main interaction processes were the formation of electron–positron pairs, the formation of which can generate secondary radiation, which can also have a negative effect. In this case, a change in the glass composition led to an increase in absorption.

**Figure 7:**
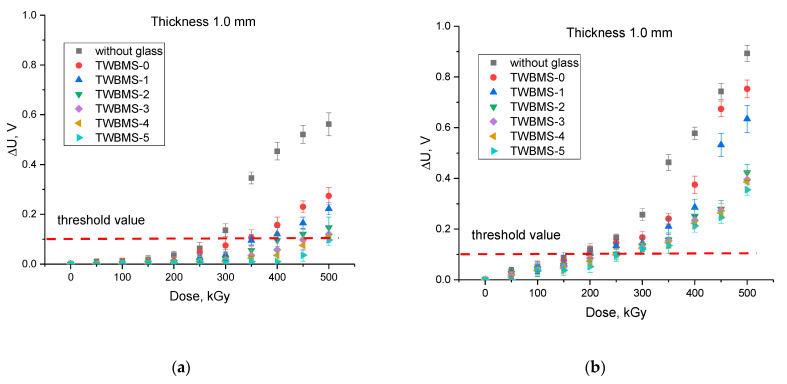
The results of changing the Δ*U* value depending on the radiation dose when shielding various types of ionizing radiation: (**a**) shielding of gamma radiation; (**b**) shielding of electronic radiation (the dotted line indicates the permissible threshold value of the ∆*U* value deviation, which is considered critical in assessing the preservation of the operability of microcircuits).

Figure 7 shows the results of assessing the change in the value of ∆*U* when measuring the current–voltage characteristics of microcircuits exposed to irradiation with gamma quanta and electrons with different doses. As can be seen from the data presented, an increase in the irradiation dose led to an increase in ∆*U*, which indicated the cumulative effect of radiation damage caused by irradiation and subsequent processes of interaction of gamma quanta and electrons with the structure of microcircuits. In the case of irradiated microcircuits without the use of protective glasses, it can be seen that when irradiated with gamma rays, the greatest damage associated with disordering and destruction was observed at radiation doses above 300 kGy. At the same time, an increase in the radiation dose above 400 kGy did not lead to significant damage or a significant increase in ∆*U*.

When using protective glasses, it was found that the excess of the threshold value ∆*U* = 0.1 V for the TWBMS–1 and TWBMS–2 samples was observed at radiation doses above 400 kGy, and at the maximum radiation dose of 500 kGy, the ∆*U* value is no more than 0.2–0.25 V, exceeding the threshold value by 1–1.5 times. For the TWBMS–3 and TWBMS–4 samples, the ∆*U* excess was no more than 0.13–0.15 V at doses of 450–500 kGy, and the ∆*U* excess point occurred at radiation doses above 400 kGy. For the TWBMS–5 samples, the excess of ∆*U* was no more than 0.095 V at the maximum radiation dose. At the same time, as can be seen from the data presented, an increase in the concentrations of MoO_3_ and SiO in the composition of the glasses led not only to a decrease in the ∆*U* deviation, but also to an increase in the stability of microcircuits during long-term irradiation due to shielding of gamma radiation and their absorption. In the TWBMS–4 and TWBMS–5 samples, an increase in ∆*U* was observed only for doses above 350 kGy, which indicates that the degree of radiation-induced damage caused by irradiation was significantly lower when using these protective glasses. This also indicates that the accumulation rate of radiation defects was significantly reduced due to gamma radiation absorption by the protective glasses.

Thus, based on the results of these studies, we can conclude that the use of glasses containing MoO_3_ and SiO concentrations above 7.5% makes it possible to protect microcircuits from the negative effects of gamma radiation, as well as to reduce the rate of formation of radiation-induced damage and the degree of their accumulation. However, at high dose loads, the shielding effect decreases, which leads to partial passage of gamma radiation through the protective glass.

When shielding electron radiation, the nature of the change in the ∆*U* value depending on the radiation dose had significant differences with high-dose irradiation in comparison with irradiation with gamma quanta. In the case of irradiation with doses of 50–200 kGy, changes in ∆*U* did not exceed the threshold value, which indicates a low degree of radiation-induced damage as well as their low concentration. However, at doses above 200 kGy, an abrupt increase in ∆*U* was observed, which indicates a deterioration in the properties of microcircuits as a result of the formation of radiation defects and their accumulation. At the same time, for the TWBMS–2, TWBMS–3, TWBMS–4, and TWBMS–5 glasses, the dependences of the changes in ∆*U* on the irradiation dose had insignificant differences, which indicates that in shielding of electron radiation, changing the composition of the glass components did not play a significant role, as was observed when shielding gamma radiation. In contrast to gamma radiation, with electron irradiation of microcircuits without protective shields, an increase in the radiation dose led to an almost linear dependence of the change in the ∆*U* value on the radiation dose, and the accumulation effect associated with a decrease in the rate of change in ∆*U* at high radiation doses, observed when shielding gamma radiation, was not observed. At the same time, at irradiation doses above 250 kGy, a decrease in the magnitude of changes in ∆*U* by more than 1.5 times was observed for samples TWBMS–3, TWBMS–4, and TWBMS–5, which indicates an effective reduction in radiation damage caused by irradiation. 

Such a difference in the nature of changes in the ∆*U* value depending on the radiation dose for different types of radiation is associated with differences in the types of radiation effects on the material. In the case of gamma radiation, the main effects and processes associated with changes in material properties are characterized by electromagnetic (the occurrence of parasitic interference or failures in the capacitance) or ionization processes. In this case, ionization processes can lead to the breaking of valence bonds or heating due to excitation processes without the subsequent formation of bias electrons. At the same time, ionization effects lead to the formation of areas with accumulated charge in the structure of the material due to the formation of free charge carriers and their further migration through the structure. The presence of such areas can lead to breakdowns, short circuits, or a decrease in the mobility of charge carriers, which leads to an increase in resistance at low radiation doses or failure at high doses. In the case of electron irradiation, ionization effects are supplemented by displacement effects associated with the formation of radiation-induced defects and their accumulation. At the same time, these effects have a strongly pronounced dependence on the radiation dose. 

**Figure 8:**
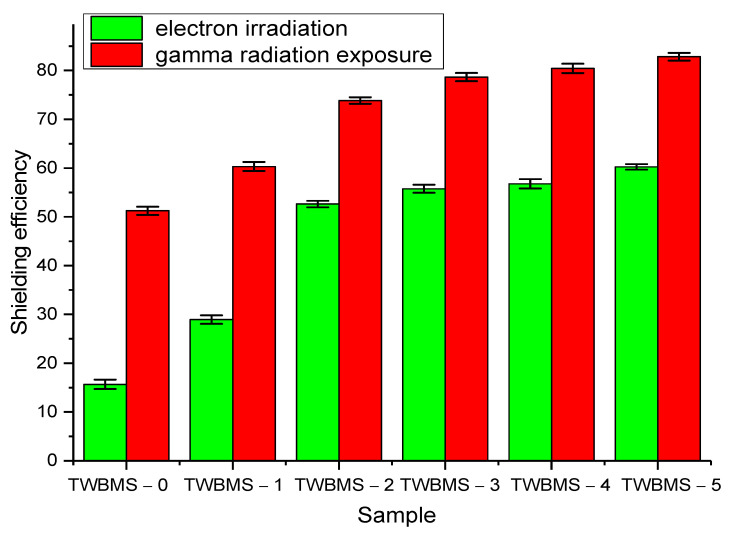
The results of a comparative analysis of the shielding efficiency when measuring the CVC at the maximum radiation dose (500 kGy).

Figure 8 shows the results of a comparative analysis of the shielding of gamma and electron radiation at a maximum radiation dose of 500 kGy in comparison with the ∆*U* value measured for microcircuits without protective shields.

As can be seen from the presented data, the maximum shielding efficiency at high radiation doses was more than 70%, which indicates that the degree of radiation damage that caused an increase in resistance and a change in the ∆U value when using protective glasses was reduced by more than three times when using TWBMS–3, TWBMS–4 and TWBMS–5 glasses. At the same time, there were no significant differences for these glasses with increases in the concentrations of MoO_3_ and SiO. In determining the shielding efficiency of electron radiation, the maximum efficiency was found to be no more than 60–65, while, as in shielding gamma radiation, an increase in the concentrations of MoO_3_ and SiO above 7.5% did not lead to a significant increase in the shielding efficiency. 

Finally, it should be noted that in recent years, the research and development of various combinations of glass, polymer, and nanoparticle compositions for radiation protection has become a highly topical, and as a result, many different glass combinations have been successfully synthesized and evaluated [41,42,43,44,45,46,47,48,49,50,51,52,53,54,55,56,57,58].

**Figure 9:**
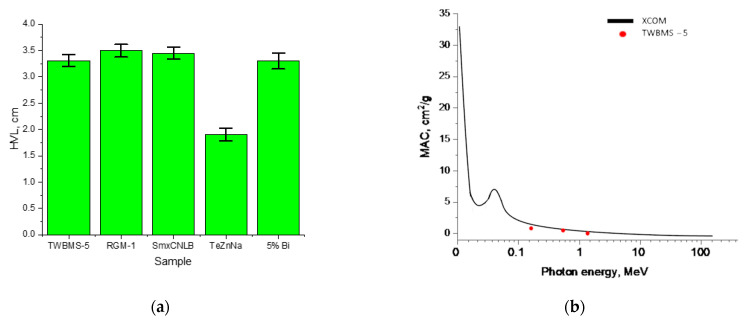
(**a**) Results of a comparative analysis of the HVL value for different types of glasses (RGM–1–a sample of soda-lime-silica glass doped with MoO_3_ [40]; SmxCNLB glasses–glass samples doped with Sm_2_O_3_ [47]; TeZnNa–tellurite glass system containing ZnO and Na_2_O [33]; 5%Bi_2_O_3_–65%P_2_O_5_–5%B_2_O_3_–25%Na_2_O [27]); (**b**) results of comparison of MAC values calculated using the XCOM program code and experimentally obtained values for the TWBMS–5 glass composition.

Figure 9a presents the results of a comparative analysis of the shielding characteristics of the TWBMS–5 sample with other types of materials, the results of which were taken from the sources [27,33,40,47]. The half-value layer (HVL) was chosen as a comparative characteristic, which makes it possible to compare the shielding characteristics of different materials. As a comparison, we chose the HVL value obtained for various glasses at a gamma-ray energy of 660 keV.

As can be seen from the presented data, the HVL value for the TWBMS–5 samples in most cases was slightly lower (by 5–7%) compared to other selected samples for comparison. However, in the case of TeZnNa samples [33], the HVL value is much lower than similar indicators, which may be due to the shielding characteristics and high absorption capacity reported by the authors in [33]. 

Figure 9b presents the results of a comparative analysis of the MAC value simulation obtained using the XCOM code and the experimentally obtained MAC values for the TWBMS–5 sample, which showed the best shielding performance among all obtained samples. As can be seen from the presented data, the results obtained are in good agreement with each other.

Thus, analyzing the results of a comparative analysis, we can conclude that the synthesized samples have rather great prospects in the field of shielding materials for protection against the negative effects of ionizing radiation.

**Figure 10:**
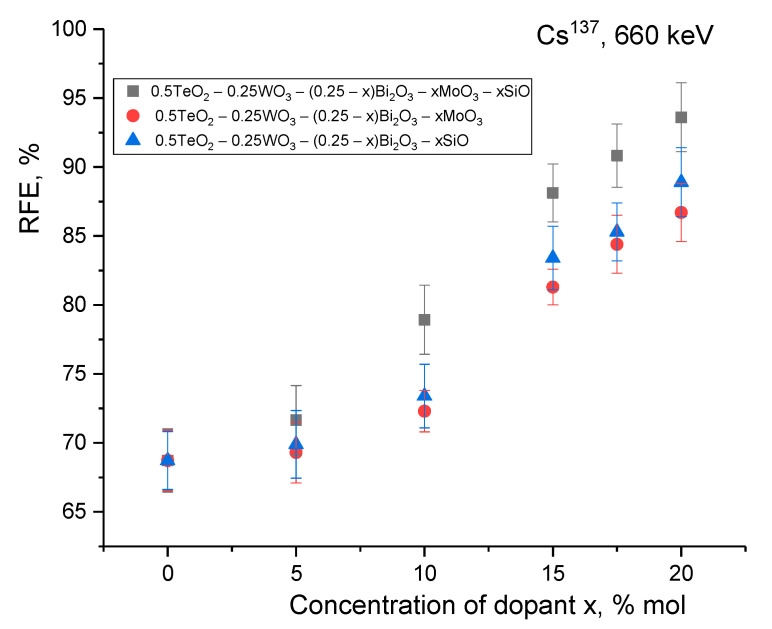
Results of RFE calculations depending on glass thickness 1.0 mm.

Figure 10 presents the results of a comparative analysis of the screening efficiency of ionizing radiation with an energy of 660 keV for glasses in which only one variation of the MoO_3_ and SiO components was used in comparison with the cases when MoO_3_ and SiO were simultaneously added to the glass composition. The purpose of this comparative analysis was to determine the contribution of each component to shielding efficiency, as well as to determine the synergistic effect associated with the addition of MoO_3_ and SiO. The shielding efficiency was evaluated for glasses with a thickness of 1 mm.

As can be seen from the presented data, the doping of 0.5TeO_2_–0.25WO_3_–0.25Bi_2_O_3_ glasses with MoO_3_ and SiO separately led to an increase in the gamma-ray shielding efficiency. The difference in screening for MoO_3_ and SiO was no more than 1.5–2.0% at the same dopant concentration. However, when MoO_3_ and SiO were used together as glass dopants, the screening efficiency increased by more than 5–10% compared to the cases of doping with only one of the MoO_3_ and SiO components. These results show that the shielding efficiency had a pronounced dependence not only on the concentration of dopants, but also on their quantity, the variation of which played a very important role in determining the absorbing and shielding characteristics.

In the future, part of the research will be aimed at studying these effects in more detail with an emphasis on studying the structural and strength characteristics of glasses.

## 4. Conclusions

In conclusion, we can summarize the intermediate results of this study. During the experiments, it was found that the addition of MoO_3_ and SiO to the composition of glasses led to an increase in the efficiency of shielding of gamma radiation with energies in the range of 130–1270 keV. At the same time, a change in the glass thickness in combination with a change in the concentrations of the MoO_3_ and SiO components was most effective in shielding of high-energy gamma rays. At the same time, for shielding low-energy gamma rays, the optimal thicknesses were no more than 1.0–1.5 mm, which, in the case of microelectronic devices, would not significantly affect their overall dimensions or increase the device weight, which are critical parameters for devices used in outer space.

Evaluating the dose dependence of the change in the ∆*U* value of microcircuits for protection against the negative effects of ionizing radiation using 1.0 mm thick protective glasses, it was found that at doses below 300 kGy, the degree of radiation damage caused by irradiation did not significantly affect the degradation of the conductive properties of microcircuits. At the same time, when using multicomponent glasses for gamma radiation shielding, the protection efficiency was more than 80–90% at maximum radiation doses.

Further research plans in this direction include conducting systematic studies to determine the resistance of these glasses to mechanical stress, as well as thermal heating, which could lead to structural changes in the properties of glasses, as well as affect the shielding efficiency.

## Figures and Tables

**Table 1:** Component content data.

Designation	Element Concentration					Stoichiometric Formula	Density, g/cm^3^
	TeO_2_	WO_3_	Bi_2_O_3_	MoO_3_	SiO		
TWBMS–0	50%	25%	25%	-	-	0.5TeO_2_ − 0.25WO_3_ − 0.25Bi_2_O_3_	6.83
TWBMS–1	50%	25%	20%	2.5%	2.5%	0.5TeO_2_ − 0.25WO_3_ − 0.20Bi_2_O_3_ − 0.025MoO_3_ − 0.025SiO	6.54
TWBMS–2	50%	25%	15%	5%	5%	0.5TeO_2_ − 0.25WO_3_ − 0.15Bi_2_O_3_ − 0.05MoO_3_ − 0.005SiO	6.25
TWBMS–3	50%	25%	10%	7.5%	7.5%	0.5TeO_2_ − 0.25WO_3_ − 0.10Bi_2_O_3_ − 0.075MoO_3_ − 0.075SiO	6.04
TWBMS–4	50%	25%	7.5%	8.75%	8.75%	0.5TeO_2_ − 0.25WO_3_ − 0.075Bi_2_O_3_ − 0.0875MoO_3_ − 0.0875SiO	5.89
TWBMS–5	50%	25%	5%	10%	10%	0.5TeO_2_ − 0.25WO_3_ − 0.05Bi_2_O_3_ − 0.1MoO_3_ − 0.1SiO	5.72

## Data Availability

Not applicable.
